# Avoiding pitfalls when combining multiple imputation and propensity scores

**DOI:** 10.1002/sim.8355

**Published:** 2019-09-11

**Authors:** Emily Granger, Jamie C. Sergeant, Mark Lunt

**Affiliations:** ^1^ Centre for Epidemiology Versus Arthritis, Division of Musculoskeletal and Dermatological Sciences The University of Manchester Manchester UK; ^2^ Centre for Biostatistics, Division of Population Health, Health Services Research and Primary Care The University of Manchester Manchester UK

**Keywords:** confounding, missing data, multiple imputation, observational data, propensity scores, simulation study

## Abstract

Overcoming bias due to confounding and missing data is challenging when analyzing observational data. Propensity scores are commonly used to account for the first problem and multiple imputation for the latter. Unfortunately, it is not known how best to proceed when both techniques are required. We investigate whether two different approaches to combining propensity scores and multiple imputation (Across and Within) lead to differences in the accuracy or precision of exposure effect estimates. Both approaches start by imputing missing values multiple times. Propensity scores are then estimated for each resulting dataset. Using the Across approach, the mean propensity score across imputations for each subject is used in a single subsequent analysis. Alternatively, the Within approach uses propensity scores individually to obtain exposure effect estimates in each imputation, which are combined to produce an overall estimate. These approaches were compared in a series of Monte Carlo simulations and applied to data from the British Society for Rheumatology Biologics Register. Results indicated that the Within approach produced unbiased estimates with appropriate confidence intervals, whereas the Across approach produced biased results and unrealistic confidence intervals. Researchers are encouraged to implement the Within approach when conducting propensity score analyses with incomplete data.

## 1 INTRODUCTION

Observational studies are useful for studying comparative effectiveness or safety of treatments, although they are prone to bias due to confounding and missing data.^1^, ^2^ Confounding can arise if variables that predict the outcome of interest also predict who is exposed to treatment: observed differences in the outcome between exposed and unexposed subjects will be partly due to differences in the distributions of these variables.^3^ Recent years have seen a marked increase in the use of propensity score (PS) methods to deal with confounding.^1^, ^4^, ^5^ The PS is defined as the probability of treatment conditional on observed baseline variables.^3^ Rosenbaum and Rubin^6^ demonstrated that by conditioning on the PS, the distribution of observed baseline variables will be balanced between exposed and unexposed groups. Using this property, the effect of confounders is reduced and unbiased estimates of the effects of exposure can be obtained.

When data are missing, it is not clear how best to implement PS methods to avoid bias and loss of precision in exposure effect estimates.^7^ A well‐established method for handling missing data is multiple imputation.^8^, ^9^, ^10^ This is a popular approach to missing data, recommended by previous studies^11^, ^12^, ^13^ demonstrating the superiority of multiple imputation compared to some of its alternatives. Usually, multiple imputation consists of three steps. Each missing value is imputed multiple times, resulting in multiple complete datasets. Each dataset is then analyzed individually and the results are pooled to obtain overall results.

Given that missing data and confounding are commonly faced challenges in analyzing observational data, where multiple imputation is often used to address the former and PSs are becoming increasingly popular for the latter, researchers may often require both techniques in their analysis. Unfortunately, combining multiple imputation with PSs is not straightforward.^14^

Mitra and Reiter^15^ described two approaches to combining multiple imputation and PSs, the Across approach and the Within approach. Both approaches start by imputing the missing values *m* times, resulting in *m* imputed datasets. PSs are then estimated in each dataset. In the Across approach, the PSs are averaged over the *m* imputed datasets to obtain an average PS. The average PS is then used in one of the imputed datasets to obtain one estimate of the effect of exposure. Alternatively, the Within approach uses the *m* PSs individually to obtain *m* exposure effect estimates. These estimates are then combined using Rubin's rules. A third approach has been suggested,^16^ in which an average PS model is obtained from the imputed datasets, by averaging the regression coefficients rather than the PSs. A single PS is then estimated using the average model, and this is used to obtain an effect estimate. As far as we know, the third approach has not been used in real analyses, unlike the Across and Within approaches.^17^ We speculate that the Within approach is used because it is more intuitive to those familiar with multiple imputation, whereas the Across approach is used because it is the least computationally intensive and requires the least data storage space. Furthermore, the Across approach is automatically implemented when using the “mi impute” command in Stata. For these reasons, we focus on the Across and Within approaches in this study.

Four prior simulation studies^13^, ^14^, ^15^, ^16^ have compared the Across and Within approaches; however, there was no consensus regarding which approach, if either, is more appropriate. In the first of these studies,^13^ the multiple imputation approaches were compared to three alternative techniques for handling missing data before using PSs. Results demonstrated that the multiple imputation approaches outperformed all three alternatives in terms of reducing bias; however, it was unclear whether either of the multiple imputation approaches outperformed the other. In the more recent studies, results either demonstrated negligible difference in the performance of the two approaches,^14^ less bias in the Across approach,^15^ or more bias in the Across approach.^16^

This research aims to aid clarification regarding the comparative performances of the Across and Within approaches. Previous research studies^13^, ^15^ have focused on the accuracy of each approach; our work builds upon this by also considering the precision of effect estimates. Furthermore, we compare the approaches in a range of scenarios not previously studied, each with varying proportions of missing data. Varying the proportions of missing data will shed light onto whether the amount of missing data affects the performance of each approach. Finally, we compare the approaches using a wide variety of different PS methods, whereas previous studies have only considered either matching^13^, ^15^ or inverse‐probability‐treatment weighting.^16^

The remainder of this article is divided into four sections. In Section 2, details are given on how to implement each approach. A simulation study comparing the approaches is presented in Section 3, and an illustrative example using real data is given in Section 4. Finally, in Section 5, the results are discussed and concluding remarks are made.

## 2 COMBINING MULTIPLE IMPUTATION AND PROPENSITY SCORES: ACROSS AND WITHIN APPROACHES

Usually, multiple imputation requires three stages: imputation, analysis, and pooling.^18^ Firstly, missing values are imputed *m* times by sampling from their posterior predictive distribution, conditional on the observed data.^2^ Consequently, there are multiple complete datasets, each of which are analyzed in the second stage using the analysis methods that were originally intended had the data been complete. In the third stage, results from the *m* analyses are pooled for inference using Rubin's rules.^18^

This is precisely what happens in the Within approach. A separate propensity model is fitted, and an estimate of the effect of exposure is calculated, in each imputed dataset. Standard errors (SEs) for each estimate are also obtained. These *m* individual estimates, and their SEs, are then combined using Rubin's rules to produce the overall estimate.

In the Across approach, a separate PS model is fitted in each imputed dataset as before. However, in this case, it is the PSs that are averaged across the imputed datasets, and a single average PS is calculated for each individual. Then, the effect of exposure and its associated SE are calculated using a single observation per subject, containing the observed outcome and exposure and the average PS.

It is important to note that if the exposure contained missing values, which were imputed, then different datasets would lead to different effect estimates in the Across approach. There would be no way of knowing which dataset contained exposure values closest to the true values, and hence no way to know which dataset would be most appropriate. In this case, the Across approach is not applicable. Additionally, it should be noted that the PS for a given subject will vary between imputations even if the subject has no missing data, since the logistic regression model that used to predict the PS will differ between imputations.

## 3 SIMULATION STUDY

### 3.1 Scenarios

We conducted a series of Monte Carlo simulations to investigate the performances of the Across and Within approaches under a wide range of scenarios.

The first scenario was a simple setting with one confounder (Scenario 1). Subsequent scenarios increased in complexity and were designed to assess the effect of: increasing the number of confounders (Scenario 2), including correlated confounders (Scenario 3), changing the direction of the association between confounder and treatment assignment (Scenario 4), and reducing the strength of the association between the confounders and exposure from strong to moderate and weak (Scenario 5 and Scenario 6, respectively). Scenarios 1‐6 all had continuous outcomes and data was simulated to be missing at random. The baseline data in Scenario 6 was used in two additional scenarios where the type of outcome and type of missing data varied; Scenario 7 had a binary outcome and data missing at random and Scenario 8 had a continuous outcome and data missing not at random.

### 3.2 Data simulation

Each scenario consisted of 1000 datasets of size 1000. Let **x**_*i*_=(*x*_1*i*_,*x*_2*i*_,…,*x*_*pi*_) denote the set of baseline covariates for the *i*th subject, **β**=(*β*_1_,*β*_2_,…,*β*_*p*_) denote the effects of covariates on outcome, *z*_*i*_ denote exposure for the *i*th subject, and **α**=(*α*_1_,*α*_2_,…,*α*_*p*_) denote the effect of covariates on exposure. The number of covariates ( *p*),**x**_*i*_,**β**, and **α** varied across scenarios and scenario specific values are given in Table 1.

**Table 1:** Details on the distribution of covariates and the effects each covariate had on the outcome and exposure in Scenarios 1‐6

	Covariates, **X**	Distribution of covariates^a^	Effect of covariates	Confounders	Effect of confounders
			on outcome, **β**		on exposure, **α**
S1	(*X*_1_,*X*_2_)	*X*_1_,*X*_2_∼*N*(0,1)	(−1,1)	(*X*_2_)	(ln(2))
S2	(*X*_1_,*X*_2_,*X*_3_,*X*_4_)	*X*_1_,*X*_2_,*X*_3_,*X*_4_∼*N*(0,1)	(−1,1,−1,−1)	(*X*_2_,*X*_3_,*X*_4_)	(ln(2),ln(2),ln(2))
S3	(*X*_1_,*X*_2_,*X*_3_,*X*_4_)	*X*_1_∼*N*(0,1)	(−1,1,−1−1)	(*X*_2_,*X*_3_,*X*_4_)	(ln(2),ln(2),ln(2))
		(*X*_2_,*X*_3_,*X*_4_)∼*MVN*(**μ**,**Σ**)			
S4	(*X*_1_,*X*_2_,*X*_3_,*X*_4_)	*X*_1_∼*N*(0,1)	(−1,1,−1,−1)	(*X*_2_,*X*_3_,*X*_4_)	(ln(2),−ln(2),ln(2))
		(*X*_2_,*X*_3_,*X*_4_)∼*MVN*(**μ**,**Σ**)			
S5	(*X*_1_,*X*_2_,*X*_3_,*X*_4_)	*X*_1_∼*N*(0,1)	(−1,1,−1.−1)	(*X*_2_,*X*_3_,*X*_4_)	(ln(1.65),ln(1.65),ln(1.65))
		(*X*_2_,*X*_3_,*X*_4_)∼*MVN*(**μ**,**Σ**)			
S6	(*X*_1_,*X*_2_,*X*_3_,*X*_4_)	*X*_1_∼*N*(0,1)	(−1,1,−1,−1)	(*X*_2_,*X*_3_,*X*_4_)	(ln(1.25),ln(1.25),ln(1.25))
		(*X*_2_,*X*_3_,*X*_4_)∼*MVN*(**μ**,**Σ**)			

a **μ**=(0,0,0) and **Σ** has variances equal to 1 with correlation 0.5. Scenarios 7‐8 used baseline data from Scenario 6.

Continuous outcomes for the *i*th subject were simulated using the following model:

(1) $$Y_{i}=\beta_{0}+\beta_{1}x_{1i}+\beta_{2}x_{2i}+\text{⋯}+\beta_{p}x_{pi}+2z_{i}+\epsilon_{i},$$

whereas binary outcomes were simulated from a Bernoulli distribution with a probability defined by:

(2) $$\text{logit}(\;p_{Yi})=\ln(\beta_{0})+\ln(\beta_{1})x_{1i}+\ln(\beta_{2})x_{2i}+\text{⋯}+\ln(\beta_{p})x_{pi}+\ln(2)z_{i}.$$

The value of *β*_0_ was selected to ensure that approximately half the subjects observed the outcome in each dataset. Let *β*_*z*_ denote the true exposure effect. For linear outcomes, *β*_*z*_=2 (Equation 1). For scenarios with binary outcomes, two potential outcomes were generated for each subject (under exposed and unexposed conditions) and these were used to calculate the true risk ratio using a method described by Austin and Stuart.^19^ For each subject, exposure was determined by simulating from the Bernoulli distribution, where the probability of the *i*th subject being exposed, *p*_*Zi*_, was defined by:

$$\text{logit}(\;p_{Zi})=\alpha_{0}+\alpha_{2}x_{2i}+\text{⋯}+\alpha_{p}x_{pi}.$$

Each scenario was repeated twice with different proportions of exposed subjects (50% and 10%). The value of *α*_0_ varied depending on the approximate number of exposed subjects required. Note that covariates *X*_2_,…,*X*_*p*_ appear in both the outcome and treatment models, hence, these were confounders. The covariate *X*_1_ was associated with the outcome only.

To investigate whether the amount of missing data affected the performance of the approaches, a variety of missing data rates were simulated in each scenario: 10%, 25%, 30%, 35%, 50%, 75%. We simulated data missing from the confounders only, because it is these values that would be used to estimate the PSs. Since multiple imputation assumes that data are missing at random (ie, the probability of missingness depends on the observed covariates), we simulated data missing at random in most scenarios. Since data was simulated missing from confounders, the only complete observed covariate was *X*_1_. Let *x*_*ji*_ denote the *i*th observation of *x*_*j*_. The probability of missingness depended on the outcome *Y* and the covariate *X*_1_ according to the following logit model:

(3) $$\text{logit}(P(x_{ji}\;\text{is missing}\;|X_{1},Y))=\gamma +\ln(1.25)x_{1i}+\ln(1.75)Y_{i},$$

where the value of *γ* varied depending on the desired proportion of missing data. To determine if the *i*th entry for each confounder was missing, indicator variables for each confounder were simulated from a Bernoulli distribution with probability as defined in Equation 3.

In real analyses, the assumption of data missing at random cannot be tested since data missing at random and data missing not at random (where in the latter probability of missingness depends on unobserved covariates) are indistinguishable to the analyst. Hence, we simulated missing not at random data in S8 to investigate the performance of both approaches when this assumption does not hold.

For data missing not at random, the probability of missingness in confounder *x*_*j*_ depended on the outcome and the confounder itself

$$\text{logit}(P(x_{ji}\;\text{is missing}\;|X_{ji},Y_{i}))=\gamma +\ln(1.25)x_{ji}+\ln(1.75)Y_{i}.$$

### 3.3 Data analysis

Missing data points were imputed 10 times using a multiple imputation by chained equations approach.^2^ The outcome and all variables included in the analysis model were included in the imputation model. PSs were then estimated using logistic regression with the true confounders as independent variables.

Exposure effect estimates were obtained after weighting, matching, or stratifying on either the average PS (Across approach) or each of the 10 PSs individually (Within approach). Readers are referred elsewhere for introductory texts on PS methods.^3^, ^6^

Two weighting schemes were applied: inverse probability of treatment weighting (IPTW) and standardized mortality ratio weighting (SMRW). Each subject was assigned a weight based on their PS, following which exposure effects were estimated using a weighted regression of the outcome on exposure. In IPTW, the weight for each subject is equal to the inverse probability of them receiving the treatment they actually received.^20^ Let *e*_*i*_ and *z*_*i*_ denote the PS and exposure status for the *i*th subject (where *z*_*i*_=1 indicates the subject is exposed). Then, the IPTW weight for the *i*th subject is defined as: $w_{i}=\frac{z_{i}}{e_{i}}+\frac{\left(1-z_{i}\right)}{\left(1-e_{i}\right)}$. In SMRW, the weights are equal to $\frac{e_{i}}{1-e_{i}}$ for unexposed subjects and 1 for exposed subjects.^20^ For calculation of the SE after using IPTW, we used the estimator recommended by Lunceford and Davidian^21^ as this estimator takes into account the fact that the PS has been estimated. A similar SE estimator has not yet been developed in the context of SMRW, therefore, a robust SE estimator was used.^22^ When matching, subjects were matched on a one‐to‐one basis using greedy matching without replacement. Matches were made on the basis of having PSs within a prescribed caliper, which was calculated using a method based on Youden's distance.^23^ An estimate of the exposure effect and associated SE were obtained by regressing the outcome on exposure using matched subjects only. Stratification was done using 10 strata and the effect estimate and SE were obtained by regressing the outcome on exposure and a categorical variable indicating which strata each subject belonged to. For both matching and stratification, effect estimates and SEs were obtained using ordinary least‐squares regression.

PS matching relies on there being a sufficiently large pool of suitable unexposed subjects to select as matches. Hence, we simulated datasets with 10% exposed subjects and applied matching to these data. However, it is common practice to simulate datasets with 50% exposed subjects in simulation studies,^24^, ^25^, ^26^ and this exposure rate may be more appropriate for stratification, to reduce the risk of obtaining lower strata with little to no exposed subjects in. Therefore, we also simulated datasets with 50% exposed subjects and stratification was applied to these data. Matching and SMRW both estimate the average effect on exposed subjects, whereas IPTW and stratification estimate the average effect of exposure on the population. We do not simulate exposure heterogeneity between groups in our scenarios, so the effect on the population and the effect on the exposed are equal. However, for the sake of comparing correct estimands, SMRW was applied to data with 10% exposure rate, whereas IPTW was applied to data with 50% exposure rate.

When the outcome was continuous, linear regression was used to estimate the exposure effect, whereas for binary outcomes, an estimate of the risk ratio was obtained using Poisson regression.^27^

Let ${\hat{\beta_{z}}}^{\left(k\right)}$ denote the exposure effect estimate in the *k*th dataset. For each analysis, the average exposure effect estimate $\left(\hat{\beta_{z}}\right)$, its bias, mean square error (MSE), the Monte Carlo standard deviation (MCSD), and average estimated standard error (SE) were calculated as $\hat{\beta_{z}}=\frac{1}{1000}\sum_{k=1}^{1000}{\hat{\beta_{z}}}^{\left(k\right)}$, bias $=\hat{\beta_{z}}-\beta_{z}$, MSE $=\frac{1}{1000}\sum_{k=1}^{1000}{\left({\hat{\beta_{z}}}^{\left(k\right)}-\beta_{z}\right)}^{2}$ , MCSD $=\sqrt{\frac{1}{999}\sum_{k=1}^{1000}{\left({\hat{\beta_{z}}}^{\left(k\right)}-\beta_{z}\right)}^{2}}$, and SE $=\frac{1}{1000}\sum_{k=1}^{1000}\text{se}({\hat{\beta_{z}}}^{\left(k\right)})$. The 95% confidence interval coverage (95% CI Cov.) was also obtained as the percent of 95% confidence intervals, which contain the true value.

### 3.4 Results

**Figure 1:**
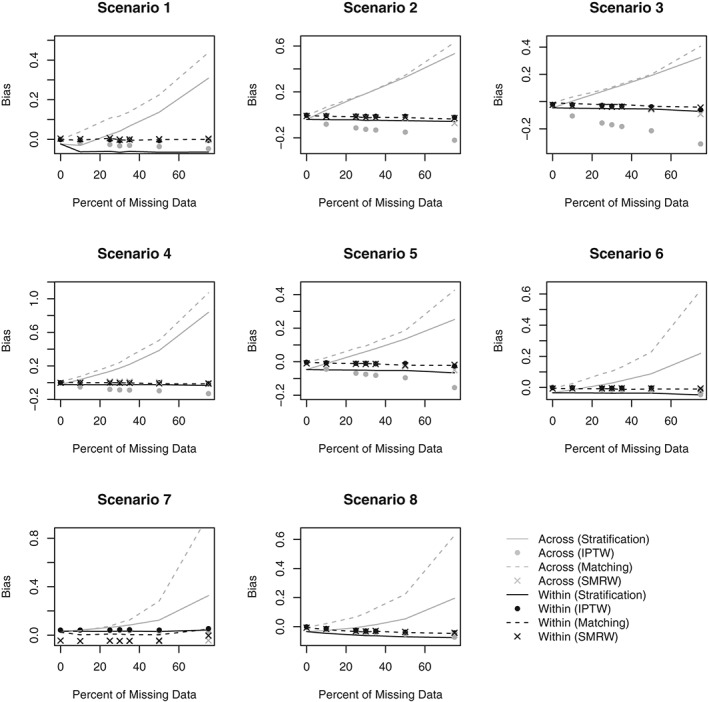
Bias in estimated exposure effect estimate by percent of missing data for Scenarios 1‐8. Scenario 1: One confounder, strong positive association with treatment, continuous outcome, data missing at random. Scenario 2: Three independent confounders, strong positive associations with treatment, continuous outcome, data missing at random. Scenario 3: Three correlated confounders, strong positive associations with treatment, continuous outcome, data missing at random. Scenario 4: Three correlated confounders, strong positive and negative associations with treatment, continuous outcome, data missing at random. Scenario 5: Three correlated confounders, moderate positive associations with treatment, continuous outcome, data missing at random. Scenario 6: Three correlated confounders, weak positive associations with treatment, continuous outcome, data missing at random. Scenario 7: Three correlated confounders, weak positive associations with treatment, binary outcome, data missing at random. Scenario 8: Three correlated confounders, weak positive associations with treatment, continuous outcome, data missing not at random. IPTW, inverse probability of treatment weighting; SMRW, standardized mortality ratio weighting

**Figure 2:**
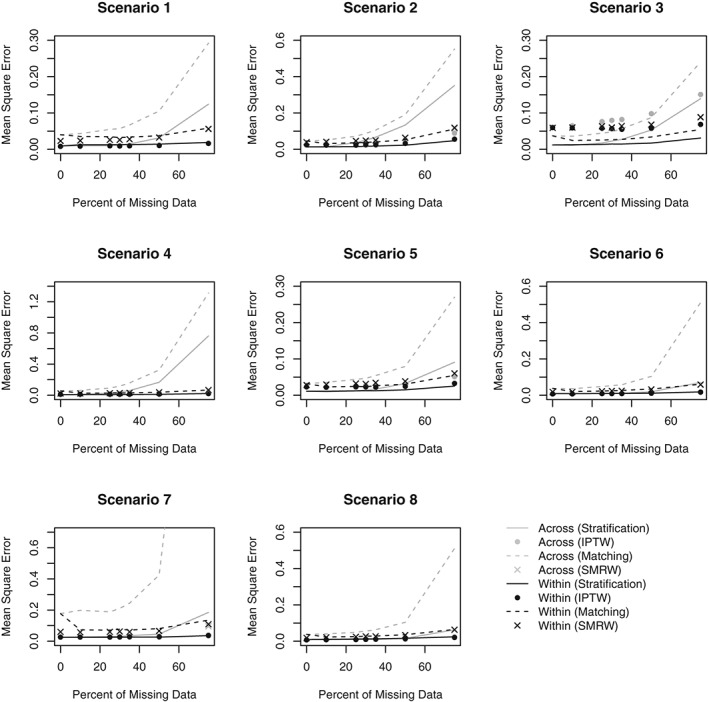
Mean square error by percent of missing data for Scenarios 1‐8. Scenario 1: One confounder, strong positive association with treatment, continuous outcome, data missing at random. Scenario 2: Three independent confounders, strong positive associations with treatment, continuous outcome, data missing at random. Scenario 3: Three correlated confounders, strong positive associations with treatment, continuous outcome, data missing at random. Scenario 4: Three correlated confounders, strong positive and negative associations with treatment, continuous outcome, data missing at random. Scenario 5: Three correlated confounders, moderate positive associations with treatment, continuous outcome, data missing at random. Scenario 6: Three correlated confounders, weak positive associations with treatment, continuous outcome, data missing at random. Scenario 7: Three correlated confounders, weak positive associations with treatment, binary outcome, data missing at random. Scenario 8: Three correlated confounders, weak positive associations with treatment, continuous outcome, data missing not at random. IPTW, inverse probability of treatment weighting; SMRW, standardized mortality ratio weighting

**Figure 3:**
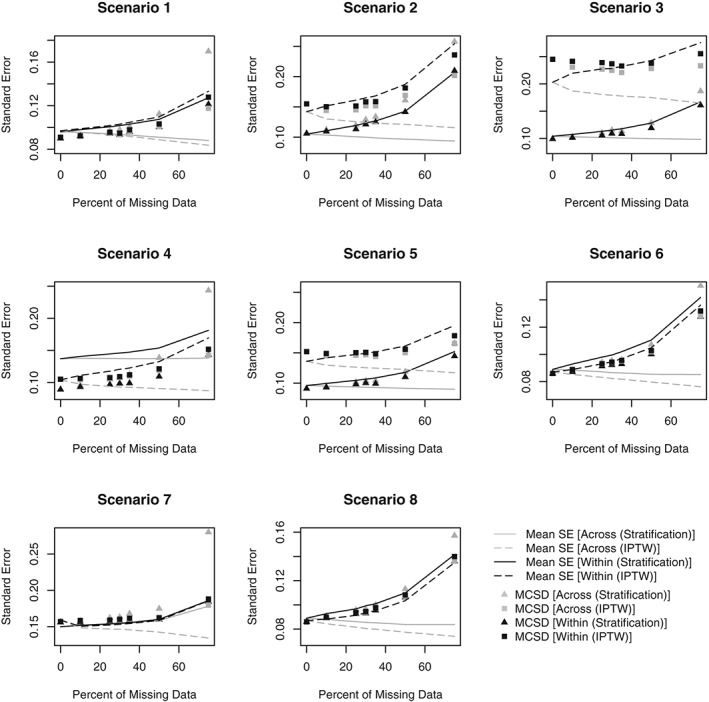
Standard error (SE) of estimate and Monte Carlo standard deviation (MCSD) by percent of missing data for Scenarios 1‐8 (for datasets with 50% prevalence of exposure). Scenario 1: One confounder, strong positive association with treatment, continuous outcome, data missing at random. Scenario 2: Three independent confounders, strong positive associations with treatment, continuous outcome, data missing at random. Scenario 3: Three correlated confounders, strong positive associations with treatment, continuous outcome, data missing at random. Scenario 4: Three correlated confounders, strong positive and negative associations with treatment, continuous outcome, data missing at random. Scenario 5: Three correlated confounders, moderate positive associations with treatment, continuous outcome, data missing at random. Scenario 6: Three correlated confounders, weak positive associations with treatment, continuous outcome, data missing at random. Scenario 7: Three correlated confounders, weak positive associations with treatment, binary outcome, data missing at random. Scenario 8: Three correlated confounders, weak positive associations with treatment, continuous outcome, data missing not at random. IPTW, inverse probability of treatment weighting

**Figure 4:**
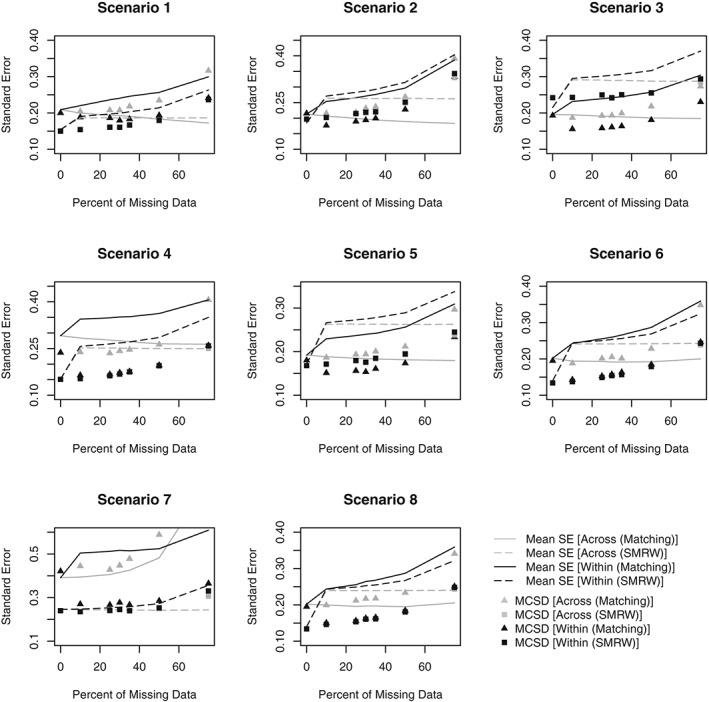
Standard error (SE) of estimate and Monte Carlo standard deviation (MCSD) by percent of missing data for Scenarios 1‐8 (for datasets with 10% prevalence of exposure). Scenario 1: One confounder, strong positive association with treatment, continuous outcome, data missing at random. Scenario 2: Three independent confounders, strong positive associations with treatment, continuous outcome, data missing at random. Scenario 3: Three correlated confounders, strong positive associations with treatment, continuous outcome, data missing at random. Scenario 4: Three correlated confounders, strong positive and negative associations with treatment, continuous outcome, data missing at random. Scenario 5: Three correlated confounders, moderate positive associations with treatment, continuous outcome, data missing at random. Scenario 6: Three correlated confounders, weak positive associations with treatment, continuous outcome, data missing at random. Scenario 7: Three correlated confounders, weak positive associations with treatment, binary outcome, data missing at random. Scenario 8: Three correlated confounders, weak positive associations with treatment, continuous outcome, data missing not at random. SMRW, standardized mortality ratio weighting

**Figure 5:**
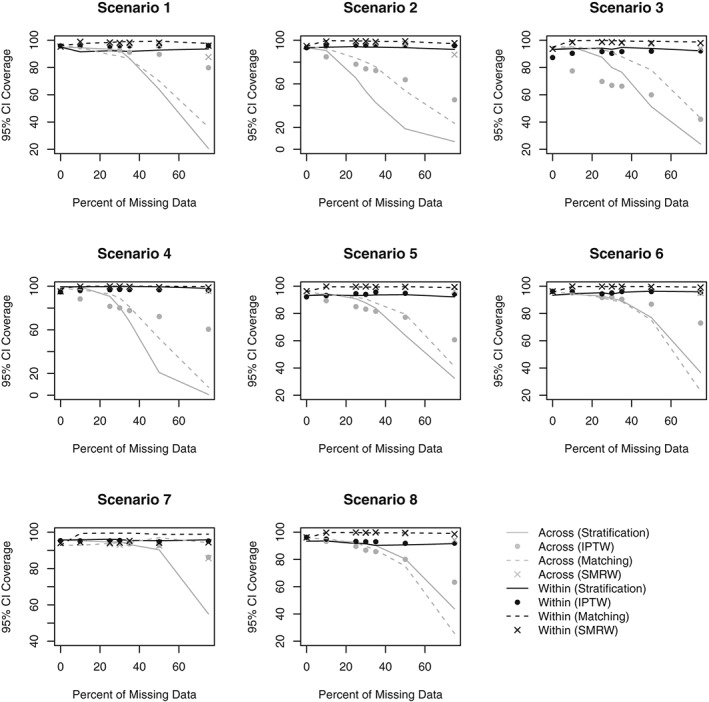
95% confidence interval (CI) coverage by percent of missing data for Scenarios 1‐8. Scenario 1: One confounder, strong positive association with treatment, continuous outcome, data missing at random. Scenario 2: Three independent confounders, strong positive associations with treatment, continuous outcome, data missing at random. Scenario 3: Three correlated confounders, strong positive associations with treatment, continuous outcome, data missing at random. Scenario 4: Three correlated confounders, strong positive and negative associations with treatment, continuous outcome, data missing at random. Scenario 5: Three correlated confounders, moderate positive associations with treatment, continuous outcome, data missing at random. Scenario 6: Three correlated confounders, weak positive associations with treatment, continuous outcome, data missing at random. Scenario 7: Three correlated confounders, weak positive associations with treatment, binary outcome, data missing at random. Scenario 8: Three correlated confounders, weak positive associations with treatment, continuous outcome, data missing not at random. IPTW, inverse probability of treatment weighting; SMRW, standardized mortality ratio weighting

Figures 1 and 2 respectively present the bias and MSE obtained in Scenarios 1‐8 for all PS analyses. Figures 3 and 4 compare the average SE and the MCSD in all scenarios for the analyses estimating exposure effects in the entire population and exposed subjects respectively. Presenting the SEs and MCSDs from all analyses in one plot made it difficult to see the trends. Finally, Figure 5 presents the 95% confidence interval coverage for all scenarios and all analyses. Tabulated results for all scenarios and analyses are presented in the online supplement.

#### 3.4.1 Bias

When using IPTW or matching on the PS, the Across approach resulted in more bias than the Within approach, regardless of the amount of missing data (Figure 1). Using SMRW, the difference in bias between methods was negligible. When stratifying on the PS, the Across approach occasionally produced less bias than the Within approach when the amount of missing data was small, but in all scenarios, the Across approach produced larger bias for missing data rates larger than 25%. At smaller missing data rates, the Across approach led to smaller bias not because the method was superior, but because there were two biases involved that canceled each other out. The negative bias was due to stratification not removing all initial confounding, this is clear since bias was observed after stratifying even when no data was missing. The negative bias affected estimates from both the Across and Within approaches. However, results indicate that the Across approach is additionally associated with a positive bias, causing the estimates to steadily increase with missing data. No such positive bias is associated with the Within approach.

#### 3.4.2 Mean square error

When matching or using IPTW, the MSE was lower in the estimates obtained using the Within approach than the Across approach. The difference in MSE when using SMRW was negligible. Stratification when the percent of missing data is 35% or less resulted in similar MSE between the two methods. However, the rate of increase in MSE with missing data was faster in the Across approach due to the observed bias (Figure 2).

#### 3.4.3 Monte Carlo standard deviation and standard error

In both approaches, regardless of PS method, the MCSD increased as missing data increased, as expected (Figures 3, 4). In the Across approach, the SEs failed to reflect the increase in variation that is demonstrated by the MCSD. Consequently, when matching, using IPTW or stratifying with the Across approach, the SEs were unrealistically small, particularly when there were large amounts of missing data. By contrast, the SEs in the Within approach increase at the same rate as the MCSD and realistic SEs were obtained when stratifying or using IPTW. Using SMRW with either method or matching with the Within approach led to inflated SEs. Matching and SMRW led to inflated SEs even when there was no missing data, suggesting that individual SE estimators were too conservative.

#### 3.4.4 95% confidence interval coverage

In all scenarios, the coverage rates in the Across approach decrease as missing data increases, falling below 95% even at small amounts of missing data (Figure 5). This is partly due to the biased estimates and partly due to the underestimation of the SEs. In the Within approach, the coverage rates remain fairly constant across all scenarios and PS methods. However, the rates obtained via stratification in the Within approach often fall just short of 95%, failing to reach adequate rates as a result of the biased estimates. The coverage rates obtained via matching in the Within approach are consistently too high; this is due to the overestimation of the SEs.

## 4 EMPIRICAL EXAMPLE USING REAL DATA

For an illustrative example, we applied both approaches to data intended to inform analysis on the long‐term safety of biologic drugs. The data is a subset of a large prospective cohort study, the British Society for Rheumatology Biologics Register (BSRBR).^28^ The current study includes rheumatoid arthritis patients who were registered in the BSRBR between 2001 and 2007. The biologic cohort includes patients receiving anti‐tumor necrosis factor (anti‐TNF) treatment, whereas patients in the comparative cohort were receiving disease‐modifying anti‐rheumatic drugs (DMARDs).

In this analysis, the Across and Within approaches were used to compare mortality in rheumatoid arthritis patients taking anti‐TNF to those taking DMARDs. However, the purpose of this analysis is to compare the approaches; not to draw valid causal inference conclusions. A PS analysis to determine whether there is an association between anti‐TNF and mortality has already been conducted on this dataset. Readers are referred to the previous analysis^29^ for a more detailed description of the data.

To demonstrate the impact of missing data, we additionally provide results from a crude analysis on the imputed data and on the complete cases (ie, patients with no missing data) only. Of the 14 697 patients included in this study, 10 967 were complete cases. A crude analysis on the complete cases obtained a risk ratio of 1.091 (95% confidence interval: 0.816, 1.459). The risk ratio obtained by a crude analysis on the imputed data was 0.938 (95% confidence interval: 0.748, 1.175). These two analyses obtain different risk ratios because the complete cases systematically differ from the patients with missing data. Failing to take these differences into account can lead to biased results. Furthermore, disregarding patients in the complete‐case analysis led to wider confidence intervals. For these reasons, a complete‐case analysis would not be appropriate. A more sophisticated missing data method, such as multiple imputation, is necessary to handle missing data in this study.

When implementing the Across and Within approaches, we included the same covariates in the PS as the previous analysis^29^; age, sex, disease activity, disability, disease duration, blood pressure, body mass index, smoking status, and information on comorbidities. Of the covariates included in the PS model, the percentage of missing data ranged from 0.014% to 11.506%.

In both approaches, results indicated that there was no significant difference in risk of mortality between patients with anti‐TNF therapies and patients taking DMARDs (Table 2). When stratifying or matching, the direction of the estimated effect changed depending on which approach is used. Using the Across approach, the estimated risk ratio after stratification was 0.778 (95% confidence interval: 0.566, 1.069) and 0.920 (95% confidence interval: 0.637, 1.330) after matching. The corresponding estimates obtained using the Within approach are 1.001 (95% confidence interval: 0.722, 1.387) and 1.049 (95% confidence interval: 0.678, 1.622). It is also worth noting that the Across approach consistently obtained narrower confidence intervals than the Within approach. While it cannot be said which approach was the least biased (because the true effect is unknown), it is likely that the confidence intervals obtained in the Within approach are more reliable. The narrow confidence intervals observed in the Across approach are due to the smaller SEs, which are less reliable since they do not take into account the between‐imputation variance. This is consistent with our simulated scenarios, where the Across approach obtained smaller SEs, often underestimating the true variance.

**Table 2:** Risk Ratios (RR), Standard Error (SE), and 95% Confidence Intervals (CI) obtained using the Across approach and the Within approach on BSRBR Data

	The Across approach			The Within approach		
	RR	SE	95% CI	RR	SE	95% CI
Stratification	0.778	0.126	0.566, 1.069	1.001	0.167	0.722, 1.387
IPTW	0.782	0.170	0.510, 1.197	0.785	0.175	0.507, 1.215
Matching	0.920	0.173	0.637, 1.330	1.049	0.230	0.678, 1.622
SMRW	0.781	0.188	0.486, 1.250	0.744	0.191	0.449, 1.230

Abbreviations: IPTW, inverse probability of treatment weighting; SMRW, standardized mortality ratio weighting.

## 5 DISCUSSION

Two methods of combining multiple imputation and PS methods, the Across approach and the Within approach, have been compared by conducting a series of Monte Carlo Simulations under a range of different scenarios.

There are a number of important trends observed in our results. Firstly, results indicated that the Across approach can produce biased estimates of the exposure effect whereas the Within approach obtained estimates with negligible bias. Secondly, the Across approach often underestimates the SE, leading to unreliable confidence intervals. Bias arises in the Across approach because, as was explained by Leyrat et al,^16^ the average PS obtained in this method is not a balancing score, whereas the estimator used in the Within approach is a true balancing score.^16^ When there is missing data, a true balancing score would need to achieve balance on both the observed and missing parts of the covariates in the original data. It is likely that the Across approach is failing to achieve balance on the missing parts, and this is why the bias increases as missing data increases. The underestimation of the SEs in the Across approach is not surprising as the method fails to take into account the between‐imputation variance. The Within approach led to more reliable SEs; however, when matching or using SMRW, the SEs were often overestimated. Given that this overestimation also occurred when there was no missing data, it is likely due to the SE estimators used in the PS methods, rather than a shortcoming of the Within approach. The implication is that, further research into appropriate SE estimators for propensity‐adjusted effect estimates would be beneficial. Additionally, we presented results comparing the Across and Within approaches in an example using real data. The real data example highlighted that the direction of exposure effect estimate may change depending on which approach to missing data is used. In this example, the conclusion would have not changed, however, in other situations, the choice of approach to handling missing data could impact the substantive conclusions of the work. In our example, the amount of missing data was relatively small. Had there been greater amounts of missing data, this may have led to greater disparity in results obtained when using different approaches.

There are four previous studies comparing the Across and Within approaches^13^, ^14^, ^15^, ^16^; however, the conclusions from these studies were contradictory. Mayer and Puschner^14^ used a complete, real dataset, and simulated missing data. Approaches were compared on the basis of how close the estimates they obtained were to the estimates obtained when data were complete. Their results indicated negligible difference in the performance of the two multiple imputation approaches. On the other hand, Mitra and Reiter^15^ compared the approaches in simulated data and their results indicated that the Within approach tends to be more biased than the Across approach. However, Penning de Vries and Groenwold^30^ demonstrated that Mitra and Reiter's results were dependent on how the imputation model was defined. They demonstrated in one of Mitra and Reiter's scenarios that omission of the outcome from the imputation model leads to greater bias reduction in the Across approach, whereas including the outcome leads to greater bias in reduction in the Within approach. In the current study, the outcome was included in the imputation model, since recent research has suggested that failing to do so may dilute the relationship between the imputed variable and the outcome.^2^, ^31^ Two additional studies^13^, ^16^ have demonstrated that including the outcome in the model leads to less bias using the Within approach in a PS matched analysis^13^ or when using inverse‐probability‐treatment‐weighting.^16^ This study adds to the evidence that the Within approach is more appropriate. Furthermore, we add to existing evidence by considering scenarios and PS methods (stratification and standardized‐mortality‐ratio‐weighting) that have not previously been investigated.

There are a number of limitations to consider. Firstly, our results may have limited generalizability since our findings rely on simulated scenarios that are not necessarily reflective of real data. A limitation of simulation studies is that the findings may be dependent upon the data generation process used. Secondly, there is a fourth PS method that we have not included in our comparisons, covariate adjustment. When using covariate adjustment, the outcome is regressed on both exposure and the PS. Covariate adjustment is the only PS method that makes strong assumptions regarding the linearity of the relationship between the PS and the outcome,^3^ and for this reason, it is often preferable to stratify, match, or assign weights.^32^ Thirdly, we did not comprehensively assess the performance of the approaches if data were missing not at random. Multiple imputation assumes that data are missing at random but it is impossible to check this assumption using the available data, hence, it may be of interest to know how the approaches perform if this assumption is violated. There are alternative methods^33^ available that have been shown to perform reliably if data are missing not at random, so researchers could consider such methods if there is a reason to believe the missing at random assumption does not hold. Fourthly, we used 10 imputations in our analyses, regardless of the amount of missing data, which is not a best practice. A recommended rule of thumb is to use a number of imputations similar to the percent of observations with missing data.^2^, ^34^ Hence, more than 10 imputations may be required, particularly when the amount of missing data is large. We chose to use 10 imputations each time to make fair comparisons between results. Finally, we were unable to accurately assess whether or not the Within approach would always obtain reliable SEs since appropriate SE estimators have not yet been developed for all PS methods. Given that in the Across approach, the SEs fail to increase with missing data, we can presume that this method would produce unreliable estimates of the SE even with appropriate estimators, however, we cannot be sure when matching or using SMRW with the Within approach.

PS methods are becoming an increasingly popular approach to handling confounding^1^ and their use may be often required when datasets are incomplete. Given that multiple imputation is a widely used method for handling missing data, it is vital that we understand how to appropriately combine multiple imputation with PSs. Results from this study indicate that the Within approach is likely to produce less biased estimates. SE estimates in both methods can be unreliable, although more so in the Across approach because this method fails to account for between‐imputation variance. Researchers are recommended to implement the Within approach when conducting a PS analysis with multiple imputation, however, care must be taken when estimating the SE.

## Supporting information

SIM_8355‐Supp‐0001‐online_supplement.docx
