# Supplementary material for: Avoiding pitfalls when combining multiple imputation and propensity scores
Source: Stat Med. 2019 Sep 11;38(26):5120–32. doi: 10.1002/sim.8355 (PMC6856837; doi:10.1002/sim.8355)
Supplement: Supplementary file 1 — SIM_8355‐Supp‐0001‐online_supplement.docx [file SIM-38-5120-s001.docx]

| Supplementary Table 1: Bias, MSE, MCSD, SE and 95% Confidence Interval Coverage for Datasets With Approximately 50% Treated Patients in Scenario 1. | | | | | | | | | | |
| --- | --- | --- | --- | --- | --- | --- | --- | --- | --- | --- |
|  | Across Approach | | | | | Within Approach | | | | |
| % missing data in confounders | Bias | MSE | MCSD | SE | 95% CI Coverage | Bias | MSE | MCSD | SE | 95% CI Coverage |
|  | *Stratification* | | | | | | | | | |
| 0^a^ | -0.023 | 0.009 | 0.090 | 0.096 | 95.0% |  |  |  |  |  |
| 10 | 0.007 | 0.009 | 0.092 | 0.095 | 95.3% | -0.026 | 0.009 | 0.091 | 0.097 | 95.5% |
| 25 | 0.063 | 0.013 | 0.097 | 0.093 | 89.1% | -0.023 | 0.009 | 0.094 | 0.100 | 95.2% |
| 30 | 0.079 | 0.016 | 0.098 | 0.093 | 85.3% | -0.027 | 0.010 | 0.094 | 0.101 | 94.8% |
| 35 | 0.106 | 0.021 | 0.102 | 0.092 | 76.3% | -0.023 | 0.010 | 0.096 | 0.103 | 95.3% |
| 50 | 0.176 | 0.044 | 0.113 | 0.090 | 51.0% | -0.027 | 0.011 | 0.101 | 0.107 | 94.8% |
| 75 | 0.352 | 0.155 | 0.175 | 0.086 | 13.3% | -0.026 | 0.016 | 0.125 | 0.129 | 95.1% |
|  | *Inverse-probability-treatment weighting* | | | | | | | | | |
| 0^a^ | -0.001 | 0.008 | 0.091 | 0.097 | 95.8% |  |  |  |  |  |
| 10 | -0.019 | 0.009 | 0.092 | 0.096 | 95.2% | -0.003 | 0.009 | 0.092 | 0.099 | 96.0% |
| 25 | -0.026 | 0.010 | 0.095 | 0.093 | 93.4% | 0.000 | 0.009 | 0.096 | 0.102 | 95.7% |
| 30 | -0.032 | 0.010 | 0.093 | 0.093 | 92.7% | -0.005 | 0.009 | 0.095 | 0.103 | 96.2% |
| 35 | -0.030 | 0.010 | 0.096 | 0.092 | 91.3% | -0.001 | 0.010 | 0.098 | 0.105 | 95.9% |
| 50 | -0.037 | 0.012 | 0.101 | 0.089 | 89.6% | -0.006 | 0.011 | 0.103 | 0.110 | 95.8% |
| 75 | -0.047 | 0.016 | 0.118 | 0.084 | 79.8% | -0.005 | 0.016 | 0.128 | 0.133 | 96.0% |
| Abbreviation: CI, confidence interval; MCSD, Monte Carlo standard deviation; MSE, mean square error; SE, standard error  ^a^No missing data so neither approach was used | | | | | | | | | | |

| Supplementary Table 2: Bias, MSE, MCSD, SE and 95% Confidence Interval Coverage for Datasets With Approximately 10% Treated Patients in Scenario 1. | | | | | | | | | | |
| --- | --- | --- | --- | --- | --- | --- | --- | --- | --- | --- |
|  | Across Approach | | | | | Within Approach | | | | |
| % missing data in confounders | Bias | MSE | MCSD | SE | 95% CI Coverage | Bias | MSE | MCSD | SE | 95% CI Coverage |
|  | *Matching* | | | | | | | | | |
| 0^a^ | -0.001 | 0.040 | 0.200 | 0.209 | 96.0% |  |  |  |  |  |
| 10 | 0.033 | 0.044 | 0.208 | 0.205 | 94.5% | -0.005 | 0.037 | 0.194 | 0.229 | 97.3% |
| 25 | 0.115 | 0.061 | 0.218 | 0.198 | 89.0% | 0.004 | 0.036 | 0.190 | 0.246 | 98.6% |
| 30 | 0.125 | 0.063 | 0.218 | 0.196 | 86.3% | -0.004 | 0.033 | 0.183 | 0.250 | 99.1% |
| 35 | 0.147 | 0.069 | 0.219 | 0.194 | 86.0% | -0.008 | 0.035 | 0.187 | 0.256 | 98.9% |
| 50 | 0.238 | 0.110 | 0.231 | 0.186 | 70.5% | -0.004 | 0.038 | 0.194 | 0.267 | 99.4% |
| 75 | 0.448 | 0.300 | 0.316 | 0.176 | 36.4% | -0.001 | 0.059 | 0.242 | 0.307 | 97.7% |
|  | *Standardised-mortality-ratio weighting* | | | | | | | | | |
| 0^a^ | 0.004 | 0.023 | 0.150 | 0.154 | 95.3% |  |  |  |  |  |
| 10 | 0.003 | 0.024 | 0.154 | 0.186 | 98.7% | 0.001 | 0.024 | 0.154 | 0.190 | 98.9% |
| 25 | 0.009 | 0.026 | 0.161 | 0.186 | 97.8% | 0.006 | 0.026 | 0.161 | 0.197 | 98.4% |
| 30 | 0.001 | 0.026 | 0.161 | 0.186 | 97.5% | -0.002 | 0.026 | 0.161 | 0.199 | 98.1% |
| 35 | 0.002 | 0.028 | 0.167 | 0.186 | 97.0% | -0.001 | 0.028 | 0.167 | 0.203 | 98.3% |
| 50 | 0.003 | 0.032 | 0.180 | 0.186 | 96.5% | 0.001 | 0.032 | 0.180 | 0.214 | 98.3% |
| 75 | -0.003 | 0.055 | 0.235 | 0.186 | 87.7% | -0.003 | 0.056 | 0.237 | 0.263 | 96.0% |
| Abbreviation: CI, confidence interval; MCSD, Monte Carlo standard deviation; MSE, mean square error; SE, standard error  ^a^No missing data so neither approach was used | | | | | | | | | | |

| Supplementary Table 3: Bias, MSE, MCSD, SE and 95% Confidence Interval Coverage for Datasets With Approximately 50% Treated Patients in Scenario 2. | | | | | | | | | | |
| --- | --- | --- | --- | --- | --- | --- | --- | --- | --- | --- |
|  | Across Approach | | | | | Within Approach | | | | |
| % missing data in confounders | Bias | MSE | MCSD | SE | 95% CI Coverage | Bias | MSE | MCSD | SE | 95% CI Coverage |
|  | *Stratification* | | | | | | | | | |
| 0^a^ | -0.040 | 0.013 | 0.106 | 0.105 | 93.1% |  |  |  |  |  |
| 10 | 0.041 | 0.014 | 0.112 | 0.104 | 90.6% | -0.042 | 0.014 | 0.109 | 0.111 | 93.6% |
| 25 | 0.151 | 0.037 | 0.120 | 0.101 | 65.4% | -0.043 | 0.015 | 0.113 | 0.119 | 94.2% |
| 30 | 0.184 | 0.051 | 0.129 | 0.100 | 53.2% | -0.048 | 0.017 | 0.121 | 0.123 | 93.7% |
| 35 | 0.220 | 0.066 | 0.134 | 0.099 | 42.9% | -0.047 | 0.018 | 0.125 | 0.127 | 93.7% |
| 50 | 0.328 | 0.133 | 0.161 | 0.097 | 18.8% | -0.051 | 0.023 | 0.142 | 0.143 | 93.2% |
| 75 | 0.533 | 0.351 | 0.258 | 0.094 | 7.1% | -0.058 | 0.047 | 0.210 | 0.207 | 91.6% |
|  | *Inverse-probability-treatment weighting* | | | | | | | | | |
| 0^a^ | -0.003 | 0.024 | 0.155 | 0.142 | 93.1% |  |  |  |  |  |
| 10 | -0.082 | 0.027 | 0.144 | 0.130 | 84.8% | -0.006 | 0.023 | 0.150 | 0.152 | 95.0% |
| 25 | -0.115 | 0.034 | 0.145 | 0.126 | 78.1% | -0.008 | 0.023 | 0.151 | 0.161 | 95.5% |
| 30 | -0.126 | 0.039 | 0.152 | 0.125 | 73.7% | -0.013 | 0.025 | 0.158 | 0.165 | 95.4% |
| 35 | -0.131 | 0.040 | 0.152 | 0.123 | 72.2% | -0.014 | 0.025 | 0.159 | 0.168 | 95.9% |
| 50 | -0.151 | 0.051 | 0.169 | 0.121 | 63.9% | -0.014 | 0.033 | 0.181 | 0.187 | 95.9% |
| 75 | -0.220 | 0.089 | 0.202 | 0.116 | 45.4% | -0.022 | 0.056 | 0.236 | 0.254 | 95.0% |
| Abbreviation: CI, confidence interval; MCSD, Monte Carlo standard deviation; MSE, mean square error; SE, standard error  ^a^No missing data so neither approach was used | | | | | | | | | | |

| Supplementary Table 4: Bias, MSE, MCSD, SE and 95% Confidence Interval Coverage for Datasets With Approximately 10% Treated Patients in Scenario 2. | | | | | | | | | | |
| --- | --- | --- | --- | --- | --- | --- | --- | --- | --- | --- |
|  | Across Approach | | | | | Within Approach | | | | |
| % missing data in confounders | Bias | MSE | MCSD | SE | 95% CI Coverage | Bias | MSE | MCSD | SE | 95% CI Coverage |
|  | *Matching* | | | | | | | | | |
| 0^a^ | -0.007 | 0.046 | 0.215 | 0.213 | 94.6% |  |  |  |  |  |
| 10 | 0.048 | 0.049 | 0.215 | 0.206 | 92.7% | -0.012 | 0.032 | 0.177 | 0.258 | 99.6% |
| 25 | 0.144 | 0.071 | 0.225 | 0.198 | 84.6% | -0.015 | 0.036 | 0.189 | 0.269 | 99.6% |
| 30 | 0.172 | 0.081 | 0.226 | 0.196 | 81.6% | -0.027 | 0.038 | 0.193 | 0.275 | 99.6% |
| 35 | 0.216 | 0.103 | 0.237 | 0.193 | 74.4% | -0.022 | 0.040 | 0.199 | 0.280 | 99.1% |
| 50 | 0.336 | 0.183 | 0.265 | 0.186 | 55.7% | -0.024 | 0.052 | 0.228 | 0.300 | 98.9% |
| 75 | 0.641 | 0.563 | 0.390 | 0.180 | 22.7% | -0.036 | 0.112 | 0.333 | 0.389 | 97.0% |
|  | *Standardised-mortality-ratio weighting* | | | | | | | | | |
| 0^a^ | -0.010 | 0.039 | 0.197 | 0.185 | 94.7% |  |  |  |  |  |
| 10 | -0.012 | 0.041 | 0.202 | 0.264 | 98.8% | -0.013 | 0.041 | 0.202 | 0.271 | 98.9% |
| 25 | -0.014 | 0.046 | 0.214 | 0.264 | 98.4% | -0.017 | 0.046 | 0.215 | 0.283 | 99.1% |
| 30 | -0.021 | 0.048 | 0.218 | 0.263 | 98.6% | -0.024 | 0.048 | 0.219 | 0.287 | 99.1% |
| 35 | -0.022 | 0.049 | 0.220 | 0.263 | 97.6% | -0.024 | 0.049 | 0.221 | 0.292 | 98.6% |
| 50 | -0.026 | 0.063 | 0.249 | 0.264 | 96.7% | -0.021 | 0.064 | 0.251 | 0.315 | 99.1% |
| 75 | -0.072 | 0.114 | 0.330 | 0.262 | 86.8% | -0.028 | 0.119 | 0.343 | 0.404 | 97.4% |
| Abbreviation: CI, confidence interval; MCSD, Monte Carlo standard deviation; MSE, mean square error; SE, standard error  ^a^No missing data so neither approach was used | | | | | | | | | | |

| Supplementary Table 5: Bias, MSE, MCSD, SE and 95% Confidence Interval Coverage for Datasets With Approximately 50% Treated Patients in Scenario 3. | | | | | | | | | | |
| --- | --- | --- | --- | --- | --- | --- | --- | --- | --- | --- |
|  | Across Approach | | | | | Within Approach | | | | |
| % missing data in confounders | Bias | MSE | MCSD | SE | 95% CI Coverage | Bias | MSE | MCSD | SE | 95% CI Coverage |
|  | *Stratification* | | | | | | | | | |
| 0^a^ | -0.046 | 0.012 | 0.099 | 0.104 | 93.2% |  |  |  |  |  |
| 10 | 0.004 | 0.011 | 0.103 | 0.103 | 95.1% | -0.048 | 0.013 | 0.101 | 0.107 | 93.8% |
| 25 | 0.073 | 0.017 | 0.110 | 0.102 | 87.6% | -0.050 | 0.014 | 0.106 | 0.113 | 94.2% |
| 30 | 0.097 | 0.023 | 0.115 | 0.101 | 80.0% | -0.052 | 0.015 | 0.109 | 0.115 | 93.4% |
| 35 | 0.119 | 0.027 | 0.114 | 0.101 | 76.6% | -0.052 | 0.014 | 0.108 | 0.118 | 94.7% |
| 50 | 0.191 | 0.053 | 0.128 | 0.100 | 51.3% | -0.054 | 0.017 | 0.119 | 0.128 | 94.0% |
| 75 | 0.323 | 0.139 | 0.187 | 0.098 | 23.8% | -0.072 | 0.031 | 0.161 | 0.167 | 92.3% |
|  | *Inverse-probability-treatment weighting* | | | | | | | | | |
| 0^a^ | -0.020 | 0.060 | 0.245 | 0.203 | 87.3% |  |  |  |  |  |
| 10 | -0.105 | 0.064 | 0.231 | 0.187 | 77.5% | -0.024 | 0.059 | 0.241 | 0.219 | 90.4% |
| 25 | -0.156 | 0.076 | 0.227 | 0.181 | 69.7% | -0.028 | 0.058 | 0.239 | 0.227 | 91.8% |
| 30 | -0.171 | 0.080 | 0.225 | 0.179 | 66.9% | -0.033 | 0.057 | 0.237 | 0.228 | 90.4% |
| 35 | -0.183 | 0.082 | 0.221 | 0.178 | 66.3% | -0.034 | 0.055 | 0.233 | 0.232 | 91.8% |
| 50 | -0.214 | 0.098 | 0.228 | 0.175 | 60.0% | -0.038 | 0.058 | 0.238 | 0.243 | 91.9% |
| 75 | -0.311 | 0.151 | 0.233 | 0.165 | 42.0% | -0.060 | 0.069 | 0.255 | 0.276 | 92.3% |
| Abbreviation: CI, confidence interval; MCSD, Monte Carlo standard deviation; MSE, mean square error; SE, standard error  ^a^No missing data so neither approach was used | | | | | | | | | | |

| Supplementary Table 6: Bias, MSE, MCSD, SE and 95% Confidence Interval Coverage for Datasets With Approximately 10% Treated Patients in Scenario 3. | | | | | | | | | | |
| --- | --- | --- | --- | --- | --- | --- | --- | --- | --- | --- |
|  | Across Approach | | | | | Within Approach | | | | |
| % missing data in confounders | Bias | MSE | MCSD | SE | 95% CI Coverage | Bias | MSE | MCSD | SE | 95% CI Coverage |
|  | *Matching* | | | | | | | | | |
| 0^a^ | -0.009 | 0.037 | 0.193 | 0.196 | 95.0% |  |  |  |  |  |
| 10 | 0.023 | 0.034 | 0.183 | 0.193 | 96.3% | -0.015 | 0.025 | 0.156 | 0.232 | 99.8% |
| 25 | 0.073 | 0.043 | 0.194 | 0.188 | 93.2% | -0.022 | 0.026 | 0.159 | 0.239 | 99.9% |
| 30 | 0.096 | 0.047 | 0.195 | 0.188 | 91.4% | -0.020 | 0.026 | 0.161 | 0.242 | 99.8% |
| 35 | 0.124 | 0.053 | 0.195 | 0.186 | 87.5% | -0.023 | 0.027 | 0.163 | 0.244 | 99.4% |
| 50 | 0.193 | 0.082 | 0.212 | 0.183 | 78.3% | -0.034 | 0.034 | 0.181 | 0.257 | 99.3% |
| 75 | 0.410 | 0.240 | 0.269 | 0.181 | 41.8% | -0.042 | 0.055 | 0.231 | 0.304 | 98.8% |
|  | *Standardised-mortality-ratio weighting* | | | | | | | | | |
| 0^a^ | -0.024 | 0.059 | 0.242 | 0.215 | 93.7% |  |  |  |  |  |
| 10 | -0.030 | 0.060 | 0.243 | 0.291 | 98.4% | -0.031 | 0.060 | 0.243 | 0.295 | 98.6% |
| 25 | -0.036 | 0.063 | 0.249 | 0.291 | 98.6% | -0.036 | 0.064 | 0.250 | 0.301 | 98.8% |
| 30 | -0.040 | 0.060 | 0.241 | 0.289 | 98.6% | -0.039 | 0.060 | 0.242 | 0.303 | 98.7% |
| 35 | -0.039 | 0.064 | 0.250 | 0.290 | 97.8% | -0.037 | 0.064 | 0.250 | 0.306 | 98.4% |
| 50 | -0.059 | 0.068 | 0.254 | 0.287 | 97.1% | -0.051 | 0.068 | 0.256 | 0.316 | 98.0% |
| 75 | -0.092 | 0.090 | 0.285 | 0.287 | 94.0% | -0.044 | 0.088 | 0.294 | 0.370 | 98.0% |
| Abbreviation: CI, confidence interval; MCSD, Monte Carlo standard deviation; MSE, mean square error; SE, standard error  ^a^No missing data so neither approach was used | | | | | | | | | | |

| Supplementary Table 7: Bias, MSE, MCSD, SE and 95% Confidence Interval Coverage for Datasets With Approximately 50% Treated Patients in Scenario 4. | | | | | | | | | | |
| --- | --- | --- | --- | --- | --- | --- | --- | --- | --- | --- |
|  | Across Approach | | | | | Within Approach | | | | |
| % missing data in confounders | Bias | MSE | MCSD | SE | 95% CI Coverage | Bias | MSE | MCSD | SE | 95% CI Coverage |
|  | *Stratification* | | | | | | | | | |
| 0^a^ | -0.023 | 0.008 | 0.089 | 0.137 | 99.5% |  |  |  |  |  |
| 10 | 0.037 | 0.011 | 0.095 | 0.138 | 99.4% | -0.024 | 0.009 | 0.093 | 0.141 | 99.5% |
| 25 | 0.134 | 0.029 | 0.105 | 0.137 | 90.7% | -0.024 | 0.010 | 0.097 | 0.144 | 99.6% |
| 30 | 0.174 | 0.042 | 0.108 | 0.137 | 80.8% | -0.025 | 0.010 | 0.098 | 0.146 | 99.7% |
| 35 | 0.219 | 0.060 | 0.113 | 0.137 | 67.6% | -0.024 | 0.010 | 0.099 | 0.147 | 99.8% |
| 50 | 0.384 | 0.167 | 0.138 | 0.137 | 20.9% | -0.023 | 0.012 | 0.109 | 0.154 | 99.5% |
| 75 | 0.838 | 0.762 | 0.243 | 0.138 | 0.7% | -0.033 | 0.022 | 0.143 | 0.181 | 98.0% |
|  | *Inverse-probability-treatment weighting* | | | | | | | | | |
| 0^a^ | 0.000 | 0.011 | 0.105 | 0.104 | 94.8% |  |  |  |  |  |
| 10 | -0.052 | 0.014 | 0.104 | 0.097 | 88.4% | 0.000 | 0.011 | 0.106 | 0.111 | 96.2% |
| 25 | -0.082 | 0.018 | 0.106 | 0.094 | 81.5% | -0.002 | 0.012 | 0.107 | 0.117 | 96.8% |
| 30 | -0.087 | 0.019 | 0.108 | 0.093 | 80.1% | -0.002 | 0.012 | 0.109 | 0.120 | 97.0% |
| 35 | -0.089 | 0.020 | 0.110 | 0.092 | 77.8% | 0.000 | 0.013 | 0.112 | 0.122 | 96.9% |
| 50 | -0.096 | 0.023 | 0.119 | 0.091 | 72.3% | 0.000 | 0.015 | 0.121 | 0.132 | 96.6% |
| 75 | -0.131 | 0.037 | 0.143 | 0.087 | 60.6% | -0.011 | 0.023 | 0.152 | 0.169 | 96.2% |
| Abbreviation: CI, confidence interval; MCSD, Monte Carlo standard deviation; MSE, mean square error; SE, standard error  ^a^No missing data so neither approach was used | | | | | | | | | | |

| Supplementary Table 8: Bias, MSE, MCSD, SE and 95% Confidence Interval Coverage for Datasets With Approximately 10% Treated Patients in Scenario 4. | | | | | | | | | | |
| --- | --- | --- | --- | --- | --- | --- | --- | --- | --- | --- |
|  | Across Approach | | | | | Within Approach | | | | |
| % missing data in confounders | Bias | MSE | MCSD | SE | 95% CI Coverage | Bias | MSE | MCSD | SE | 95% CI Coverage |
|  | *Matching* | | | | | | | | | |
| 0^a^ | 0.008 | 0.056 | 0.237 | 0.291 | 98.0% |  |  |  |  |  |
| 10 | 0.071 | 0.068 | 0.239 | 0.284 | 96.8% | -0.002 | 0.027 | 0.166 | 0.354 | 100.0% |
| 25 | 0.217 | 0.104 | 0.235 | 0.277 | 92.8% | -0.001 | 0.029 | 0.169 | 0.358 | 100.0% |
| 30 | 0.271 | 0.136 | 0.242 | 0.275 | 89.3% | -0.002 | 0.030 | 0.173 | 0.360 | 100.0% |
| 35 | 0.331 | 0.176 | 0.246 | 0.272 | 81.0% | -0.003 | 0.032 | 0.178 | 0.362 | 100.0% |
| 50 | 0.544 | 0.366 | 0.263 | 0.265 | 52.2% | -0.013 | 0.039 | 0.198 | 0.371 | 100.0% |
| 75 | 1.101 | 1.369 | 0.406 | 0.264 | 7.4% | -0.015 | 0.068 | 0.261 | 0.413 | 99.8% |
|  | *Standardised-mortality-ratio weighting* | | | | | | | | | |
| 0^a^ | -0.002 | 0.023 | 0.151 | 0.152 | 95.3% |  |  |  |  |  |
| 10 | 0.005 | 0.023 | 0.153 | 0.252 | 99.7% | 0.023 | 0.153 | 0.257 | 99.7% | 0.023 |
| 25 | 0.014 | 0.026 | 0.162 | 0.251 | 99.7% | 0.026 | 0.163 | 0.265 | 99.9% | 0.026 |
| 30 | 0.013 | 0.028 | 0.167 | 0.251 | 99.8% | 0.028 | 0.168 | 0.268 | 99.9% | 0.028 |
| 35 | 0.012 | 0.031 | 0.174 | 0.251 | 99.1% | 0.031 | 0.175 | 0.271 | 99.6% | 0.031 |
| 50 | 0.005 | 0.037 | 0.193 | 0.250 | 98.8% | 0.038 | 0.195 | 0.285 | 99.2% | 0.038 |
| 75 | -0.016 | 0.063 | 0.251 | 0.250 | 95.7% | 0.067 | 0.259 | 0.350 | 98.9% | 0.067 |
| Abbreviation: CI, confidence interval; MCSD, Monte Carlo standard deviation; MSE, mean square error; SE, standard error  ^a^No missing data so neither approach was used | | | | | | | | | | |

| Supplementary Table 9: Bias, MSE, MCSD, SE and 95% Confidence Interval Coverage for Datasets With Approximately 50% Treated Patients in Scenario 5. | | | | | | | | | | |
| --- | --- | --- | --- | --- | --- | --- | --- | --- | --- | --- |
|  | Across Approach | | | | | Within Approach | | | | |
| % missing data in confounders | Bias | MSE | MCSD | SE | 95% CI Coverage | Bias | MSE | MCSD | SE | 95% CI Coverage |
|  | *Stratification* | | | | | | | | | |
| 0^a^ | -0.470 | 0.011 | 0.091 | 0.096 | 93.1% | -0.049 | 0.011 | 0.093 | 0.100 | 93.6% |
| 10 | -0.011 | 0.009 | 0.095 | 0.095 | 94.6% | -0.050 | 0.012 | 0.098 | 0.105 | 93.1% |
| 25 | 0.043 | 0.012 | 0.101 | 0.094 | 90.8% | -0.050 | 0.013 | 0.100 | 0.107 | 93.3% |
| 30 | 0.060 | 0.014 | 0.104 | 0.093 | 87.8% | -0.050 | 0.013 | 0.099 | 0.109 | 93.6% |
| 35 | 0.078 | 0.017 | 0.104 | 0.093 | 83.9% | -0.052 | 0.015 | 0.110 | 0.118 | 93.8% |
| 50 | 0.135 | 0.032 | 0.119 | 0.092 | 64.0% | -0.066 | 0.025 | 0.145 | 0.152 | 92.2% |
| 75 | 0.252 | 0.091 | 0.166 | 0.090 | 32.5% | -0.068 | 0.056 | 0.230 | 0.253 | 93.7% |
|  | *Inverse-probability-treatment weighting* | | | | | | | | | |
| 0^a^ | -0.004 | 0.023 | 0.152 | 0.136 | 92.3% |  |  |  |  |  |
| 10 | -0.044 | 0.023 | 0.145 | 0.130 | 89.4% | -0.006 | 0.022 | 0.149 | 0.142 | 93.1% |
| 25 | -0.069 | 0.026 | 0.146 | 0.126 | 84.9% | -0.009 | 0.023 | 0.150 | 0.147 | 94.6% |
| 30 | -0.075 | 0.027 | 0.147 | 0.125 | 83.1% | -0.010 | 0.023 | 0.151 | 0.149 | 94.0% |
| 35 | -0.081 | 0.027 | 0.144 | 0.125 | 81.7% | -0.011 | 0.022 | 0.148 | 0.152 | 95.7% |
| 50 | -0.095 | 0.032 | 0.150 | 0.123 | 77.3% | -0.011 | 0.024 | 0.156 | 0.161 | 94.3% |
| 75 | -0.154 | 0.051 | 0.165 | 0.118 | 60.8% | -0.027 | 0.032 | 0.178 | 0.195 | 95.8% |
| Abbreviation: CI, confidence interval; MCSD, Monte Carlo standard deviation; MSE, mean square error; SE, standard error  ^a^No missing data so neither approach was used | | | | | | | | | | |

| Supplementary Table 10: Bias, MSE, MCSD, SE and 95% Confidence Interval Coverage for Datasets With Approximately 10% Treated Patients in Scenario 5. | | | | | | | | | | |
| --- | --- | --- | --- | --- | --- | --- | --- | --- | --- | --- |
|  | Across Approach | | | | | Within Approach | | | | |
| % missing data in confounders | Bias | MSE | MCSD | SE | 95% CI Coverage | Bias | MSE | MCSD | SE | 95% CI Coverage |
|  | *Matching* | | | | | | | | | |
| 0^a^ | -0.003 | 0.032 | 0.180 | 0.192 | 96.0 |  |  |  |  |  |
| 10 | 0.024 | 0.035 | 0.187 | 0.188 | 93.8 | -0.009 | 0.023 | 0.151 | 0.229 | 99.6% |
| 25 | 0.080 | 0.044 | 0.193 | 0.185 | 91.9 | -0.011 | 0.024 | 0.156 | 0.237 | 99.5% |
| 30 | 0.094 | 0.046 | 0.194 | 0.184 | 90.7 | -0.011 | 0.024 | 0.154 | 0.240 | 99.4% |
| 35 | 0.118 | 0.054 | 0.201 | 0.183 | 87.7 | -0.013 | 0.026 | 0.161 | 0.242 | 99.4% |
| 50 | 0.186 | 0.079 | 0.212 | 0.181 | 79.5 | -0.021 | 0.031 | 0.174 | 0.256 | 99.7% |
| 75 | 0.426 | 0.270 | 0.297 | 0.180 | 41.0 | -0.022 | 0.055 | 0.233 | 0.309 | 98.9% |
|  | *Standardised-mortality-ratio weighting* | | | | | | | | | |
| 0^a^ | -0.010 | 0.028 | 0.168 | 0.171 | 96.4% |  |  |  |  |  |
| 10 | -0.013 | 0.030 | 0.171 | 0.263 | 99.8% | -0.014 | 0.030 | 0.171 | 0.266 | 99.8% |
| 25 | -0.013 | 0.032 | 0.179 | 0.263 | 99.6% | -0.013 | 0.032 | 0.180 | 0.273 | 99.7% |
| 30 | -0.015 | 0.031 | 0.175 | 0.263 | 99.8% | -0.014 | 0.031 | 0.176 | 0.275 | 99.8% |
| 35 | -0.015 | 0.034 | 0.184 | 0.263 | 99.5% | -0.013 | 0.034 | 0.185 | 0.278 | 99.5% |
| 50 | -0.029 | 0.038 | 0.193 | 0.262 | 99.3% | -0.022 | 0.038 | 0.195 | 0.289 | 99.6% |
| 75 | -0.051 | 0.059 | 0.239 | 0.263 | 96.6% | -0.018 | 0.060 | 0.245 | 0.338 | 99.4% |
| Abbreviation: CI, confidence interval; MCSD, Monte Carlo standard deviation; MSE, mean square error; SE, standard error  ^a^No missing data so neither approach was used | | | | | | | | | | |

| Supplementary Table 11: Bias, MSE, MCSD, SE and 95% Confidence Interval Coverage for Datasets With Approximately 50% Treated Patients in Scenario 6. | | | | | | | | | | |
| --- | --- | --- | --- | --- | --- | --- | --- | --- | --- | --- |
|  | Across Approach | | | | | Within Approach | | | | |
| % missing data in confounders | Bias | MSE | MCSD | SE | 95% CI Coverage | Bias | MSE | MCSD | SE | 95% CI Coverage |
|  | *Stratification* | | | | | | | | | |
| 0^a^ | -0.034 | 0.009 | 0.086 | 0.089 | 93.4% |  |  |  |  |  |
| 10 | -0.015 | 0.008 | 0.088 | 0.088 | 94.1% | -0.035 | 0.009 | 0.087 | 0.093 | 94.5% |
| 25 | 0.017 | 0.009 | 0.094 | 0.087 | 92.8% | -0.036 | 0.010 | 0.091 | 0.098 | 95.2% |
| 30 | 0.028 | 0.010 | 0.095 | 0.087 | 90.9% | -0.037 | 0.010 | 0.092 | 0.099 | 94.2% |
| 35 | 0.043 | 0.011 | 0.096 | 0.086 | 89.5% | -0.036 | 0.010 | 0.093 | 0.102 | 95.6% |
| 50 | 0.088 | 0.019 | 0.107 | 0.085 | 77.1% | -0.037 | 0.011 | 0.100 | 0.110 | 96.3% |
| 75 | 0.219 | 0.070 | 0.150 | 0.085 | 36.7% | -0.048 | 0.019 | 0.128 | 0.142 | 95.9% |
|  | *Inverse-probability-treatment weighting* | | | | | | | | | |
| 0^a^ | -0.001 | 0.007 | 0.086 | 0.087 | 96.2% |  |  |  |  |  |
| 10 | -0.008 | 0.008 | 0.089 | 0.085 | 95.0% | -0.002 | 0.008 | 0.089 | 0.089 | 96.0% |
| 25 | -0.014 | 0.009 | 0.093 | 0.083 | 91.7% | -0.003 | 0.009 | 0.093 | 0.093 | 94.4% |
| 30 | -0.016 | 0.009 | 0.094 | 0.082 | 91.9% | -0.004 | 0.009 | 0.094 | 0.095 | 95.0% |
| 35 | -0.016 | 0.009 | 0.095 | 0.082 | 90.4% | -0.003 | 0.009 | 0.096 | 0.097 | 96.0% |
| 50 | -0.020 | 0.011 | 0.102 | 0.080 | 86.9% | -0.004 | 0.011 | 0.103 | 0.105 | 96.0% |
| 75 | -0.046 | 0.019 | 0.128 | 0.076 | 73.0% | -0.015 | 0.018 | 0.132 | 0.136 | 95.4% |
| Abbreviation: CI, confidence interval; MCSD, Monte Carlo standard deviation; MSE, mean square error; SE, standard error  ^a^No missing data so neither approach was used | | | | | | | | | | |

| Supplementary Table 12: Bias, MSE, MCSD, SE and 95% Confidence Interval Coverage for Datasets With Approximately 10% Treated Patients in Scenario 6. | | | | | | | | | | |
| --- | --- | --- | --- | --- | --- | --- | --- | --- | --- | --- |
|  | Across Approach | | | | | Within Approach | | | | |
| % missing data in confounders | Bias | MSE | MCSD | SE | 95% CI Coverage | Bias | MSE | MCSD | SE | 95% CI Coverage |
|  | *Matching* | | | | | | | | | |
| 0^a^ | -0.005 | 0.038 | 0.195 | 0.202 | 95.6% |  |  |  |  |  |
| 10 | 0.023 | 0.039 | 0.195 | 0.200 | 95.6% | -0.008 | 0.021 | 0.144 | 0.255 | 99.8% |
| 25 | 0.080 | 0.047 | 0.201 | 0.198 | 92.4% | -0.008 | 0.024 | 0.155 | 0.267 | 99.8% |
| 30 | 0.109 | 0.055 | 0.208 | 0.197 | 89.1% | -0.007 | 0.025 | 0.159 | 0.272 | 100.0% |
| 35 | 0.129 | 0.060 | 0.208 | 0.196 | 89.5% | -0.013 | 0.027 | 0.165 | 0.278 | 99.8% |
| 50 | 0.228 | 0.106 | 0.233 | 0.196 | 73.2% | -0.009 | 0.035 | 0.187 | 0.298 | 99.7% |
| 75 | 0.626 | 0.513 | 0.349 | 0.203 | 24.1% | -0.012 | 0.062 | 0.249 | 0.370 | 99.4% |
|  | *Standardised-mortality-ratio weighting* | | | | | | | | | |
| 0^a^ | -0.007 | 0.018 | 0.134 | 0.140 | 96.2% |  |  |  |  |  |
| 10 | -0.007 | 0.019 | 0.137 | 0.241 | 99.8% | -0.007 | 0.019 | 0.137 | 0.245 | 99.9% |
| 25 | -0.007 | 0.022 | 0.149 | 0.241 | 99.8% | -0.007 | 0.022 | 0.149 | 0.250 | 99.8% |
| 30 | -0.005 | 0.024 | 0.154 | 0.241 | 99.8% | -0.005 | 0.024 | 0.154 | 0.253 | 99.8% |
| 35 | -0.006 | 0.025 | 0.157 | 0.241 | 99.7% | -0.006 | 0.025 | 0.157 | 0.256 | 99.8% |
| 50 | -0.008 | 0.032 | 0.178 | 0.242 | 99.4% | -0.006 | 0.032 | 0.179 | 0.269 | 99.6% |
| 75 | -0.019 | 0.057 | 0.239 | 0.243 | 95.0% | -0.006 | 0.059 | 0.243 | 0.324 | 99.1% |
| Abbreviation: CI, confidence interval; MCSD, Monte Carlo standard deviation; MSE, mean square error; SE, standard error  ^a^No missing data so neither approach was used | | | | | | | | | | |

| Supplementary Table 13: Bias, MSE, MCSD, SE and 95% Confidence Interval Coverage for Datasets With Approximately 50% Treated Patients in Scenario 7. | | | | | | | | | | |
| --- | --- | --- | --- | --- | --- | --- | --- | --- | --- | --- |
|  | Across Approach | | | | | Within Approach | | | | |
| % missing data in confounders | Bias | MSE | MCSD | SE | 95% CI Coverage | Bias | MSE | MCSD | SE | 95% CI Coverage |
|  | *Stratification* | | | | | | | | | |
| 0^a^ | 0.032 | 0.025 | 0.156 | 0.150 | 95.7% |  |  |  |  |  |
| 10 | 0.042 | 0.027 | 0.158 | 0.151 | 95.3% | 0.032 | 0.025 | 0.156 | 0.152 | 95.8% |
| 25 | 0.064 | 0.030 | 0.162 | 0.153 | 94.6% | 0.031 | 0.026 | 0.157 | 0.154 | 96.1% |
| 30 | 0.073 | 0.032 | 0.163 | 0.154 | 94.8% | 0.033 | 0.026 | 0.157 | 0.155 | 95.9% |
| 35 | 0.084 | 0.035 | 0.168 | 0.155 | 93.2% | 0.033 | 0.026 | 0.159 | 0.156 | 95.5% |
| 50 | 0.124 | 0.046 | 0.175 | 0.159 | 90.3% | 0.030 | 0.026 | 0.160 | 0.160 | 95.3% |
| 75 | 0.326 | 0.185 | 0.280 | 0.178 | 55.0% | 0.043 | 0.036 | 0.184 | 0.186 | 95.8% |
|  | *Inverse-probability-treatment weighting* | | | | | | | | | |
| 0^a^ | 0.043 | 0.026 | 0.157 | 0.159 | 95.3% |  |  |  |  |  |
| 10 | 0.041 | 0.027 | 0.158 | 0.149 | 95.0% | 0.043 | 0.027 | 0.158 | 0.150 | 95.2% |
| 25 | 0.037 | 0.026 | 0.158 | 0.147 | 94.7% | 0.042 | 0.027 | 0.159 | 0.152 | 95.2% |
| 30 | 0.038 | 0.027 | 0.159 | 0.146 | 94.7% | 0.044 | 0.028 | 0.160 | 0.153 | 95.4% |
| 35 | 0.037 | 0.027 | 0.160 | 0.146 | 93.6% | 0.044 | 0.028 | 0.162 | 0.154 | 94.6% |
| 50 | 0.029 | 0.027 | 0.160 | 0.143 | 92.8% | 0.041 | 0.028 | 0.162 | 0.159 | 94.8% |
| 75 | 0.023 | 0.033 | 0.180 | 0.134 | 86.2% | 0.052 | 0.038 | 0.188 | 0.186 | 95.1% |
| Abbreviation: CI, confidence interval; MCSD, Monte Carlo standard deviation; MSE, mean square error; SE, standard error  ^a^No missing data so neither approach was used | | | | | | | | | | |

| Supplementary Table 14: Bias, MSE, MCSD, SE and 95% Confidence Interval Coverage for Datasets With Approximately 10% Treated Patients in Scenario 7. | | | | | | | | | | |
| --- | --- | --- | --- | --- | --- | --- | --- | --- | --- | --- |
|  | Across Approach | | | | | Within Approach | | | | |
| % missing data in confounders | Bias | MSE | MCSD | SE | 95% CI Coverage | Bias | MSE | MCSD | SE | 95% CI Coverage |
|  | *Matching* | | | | | | | | | |
| 0^a^ | 0.024 | 0.177 | 0.421 | 0.391 | 92.8% |  |  |  |  |  |
| 10 | 0.036 | 0.199 | 0.444 | 0.394 | 92.8% | 0.003 | 0.073 | 0.270 | 0.504 | 99.3% |
| 25 | 0.074 | 0.188 | 0.428 | 0.406 | 93.8% | 0.009 | 0.071 | 0.267 | 0.512 | 99.5% |
| 30 | 0.103 | 0.210 | 0.447 | 0.416 | 94.3% | 0.009 | 0.077 | 0.277 | 0.517 | 99.4% |
| 35 | 0.125 | 0.244 | 0.478 | 0.426 | 94.3% | 0.004 | 0.071 | 0.267 | 0.514 | 99.5% |
| 50 | 0.281 | 0.425 | 0.588 | 0.482 | 97.0% | 0.003 | 0.081 | 0.285 | 0.524 | 98.8% |
| 75 | 0.990 | 3.102 | 1.457 | 0.827 | 94.2% | 0.050 | 0.135 | 0.365 | 0.609 | 98.9% |
|  | *Standardised-mortality-ratio weighting* | | | | | | | | | |
| 0^a^ | -0.045 | 0.060 | 0.240 | 0.246 | 94.0% |  |  |  |  |  |
| 10 | -0.049 | 0.058 | 0.235 | 0.244 | 94.4% | -0.049 | 0.058 | 0.236 | 0.247 | 94.5% |
| 25 | -0.048 | 0.060 | 0.240 | 0.243 | 93.4% | -0.046 | 0.060 | 0.241 | 0.253 | 94.1% |
| 30 | -0.050 | 0.062 | 0.245 | 0.243 | 93.1% | -0.047 | 0.062 | 0.246 | 0.256 | 94.2% |
| 35 | -0.050 | 0.060 | 0.239 | 0.243 | 94.2% | -0.047 | 0.060 | 0.240 | 0.258 | 95.2% |
| 50 | -0.054 | 0.065 | 0.250 | 0.242 | 92.6% | -0.045 | 0.066 | 0.253 | 0.273 | 94.2% |
| 75 | -0.043 | 0.096 | 0.307 | 0.243 | 85.7% | -0.004 | 0.109 | 0.331 | 0.357 | 94.4% |
| Abbreviation: CI, confidence interval; MCSD, Monte Carlo standard deviation; MSE, mean square error; SE, standard error  ^a^No missing data so neither approach was used | | | | | | | | | | |

| Supplementary Table 15: Bias, MSE, MCSD, SE and 95% Confidence Interval Coverage for Datasets With Approximately 50% Treated Patients in Scenario 8. | | | | | | | | | | |
| --- | --- | --- | --- | --- | --- | --- | --- | --- | --- | --- |
|  | Across Approach | | | | | Within Approach | | | | |
| % missing data in confounders | Bias | MSE | MCSD | SE | 95% CI Coverage | Bias | MSE | MCSD | SE | 95% CI Coverage |
|  | *Stratification* | | | | | | | | | |
| 0^a^ | -0.034 | 0.009 | 0.086 | 0.089 | 93.4% |  |  |  |  |  |
| 10 | -0.026 | 0.009 | 0.091 | 0.088 | 93.7% | -0.046 | 0.010 | 0.089 | 0.093 | 93.5% |
| 25 | -0.007 | 0.009 | 0.096 | 0.086 | 92.8% | -0.057 | 0.012 | 0.092 | 0.097 | 91.7% |
| 30 | 0.004 | 0.009 | 0.097 | 0.086 | 91.4% | -0.062 | 0.012 | 0.093 | 0.099 | 91.5% |
| 35 | 0.014 | 0.010 | 0.100 | 0.085 | 90.2% | -0.063 | 0.013 | 0.095 | 0.101 | 90.3% |
| 50 | 0.053 | 0.016 | 0.113 | 0.084 | 80.4% | -0.070 | 0.016 | 0.106 | 0.110 | 90.7% |
| 75 | 0.195 | 0.063 | 0.157 | 0.084 | 43.%7 | -0.076 | 0.024 | 0.136 | 0.141 | 91.6% |
|  | *Inverse-probability-treatment weighting* | | | | | | | | | |
| 0^a^ | -0.001 | 0.007 | 0.086 | 0.087 | 96.2% |  |  |  |  |  |
| 10 | -0.019 | 0.008 | 0.090 | 0.084 | 93.3% | -0.012 | 0.008 | 0.090 | 0.088 | 94.9% |
| 25 | -0.035 | 0.010 | 0.093 | 0.082 | 89.7% | -0.025 | 0.009 | 0.094 | 0.091 | 93.3% |
| 30 | -0.040 | 0.010 | 0.094 | 0.081 | 86.7% | -0.029 | 0.010 | 0.095 | 0.093 | 92.9% |
| 35 | -0.042 | 0.011 | 0.097 | 0.080 | 85.8% | -0.030 | 0.010 | 0.098 | 0.095 | 93.0% |
| 50 | -0.053 | 0.014 | 0.107 | 0.077 | 80.1% | -0.038 | 0.013 | 0.108 | 0.103 | 91.8% |
| 75 | -0.072 | 0.024 | 0.136 | 0.074 | 63.2% | -0.042 | 0.021 | 0.140 | 0.135 | 91.9% |
| Abbreviation: CI, confidence interval; MCSD, Monte Carlo standard deviation; MSE, mean square error; SE, standard error  ^a^No missing data so neither approach was used | | | | | | | | | | |

| Supplementary Table 16: Bias, MSE, MCSD, SE and 95% Confidence Interval Coverage for Datasets With Approximately 10% Treated Patients in Scenario 8. | | | | | | | | | | |
| --- | --- | --- | --- | --- | --- | --- | --- | --- | --- | --- |
|  | Across Approach | | | | | Within Approach | | | | |
| % missing data in confounders | Bias | MSE | MCSD | SE | 95% CI Coverage | Bias | MSE | MCSD | SE | 95% CI Coverage |
|  | *Matching* | | | | | | | | | |
| 0^a^ | -0.005 | 0.038 | 0.195 | 0.202 | 95.6% |  |  |  |  |  |
| 10 | 0.021 | 0.040 | 0.199 | 0.200 | 95.3% | -0.021 | 0.023 | 0.150 | 0.244 | 99.8% |
| 25 | 0.066 | 0.049 | 0.212 | 0.196 | 92.0% | -0.033 | 0.026 | 0.156 | 0.256 | 99.8% |
| 30 | 0.093 | 0.056 | 0.217 | 0.197 | 89.6% | -0.036 | 0.028 | 0.163 | 0.264 | 99.5% |
| 35 | 0.126 | 0.063 | 0.217 | 0.197 | 87.0% | -0.032 | 0.028 | 0.165 | 0.268 | 99.5% |
| 50 | 0.223 | 0.104 | 0.234 | 0.195 | 75.1% | -0.040 | 0.036 | 0.184 | 0.287 | 99.5% |
| 75 | 0.627 | 0.510 | 0.341 | 0.205 | 25.7% | -0.047 | 0.065 | 0.251 | 0.359 | 99.1% |
|  | *Standardised-mortality-ratio weighting* | | | | | | | | | |
| 0^a^ | -0.007 | 0.018 | 0.134 | 0.140 | 96.2% |  |  |  |  |  |
| 10 | -0.016 | 0.022 | 0.146 | 0.240 | 99.7% | -0.016 | 0.022 | 0.146 | 0.243 | 99.7% |
| 25 | -0.030 | 0.024 | 0.153 | 0.239 | 99.8% | -0.030 | 0.024 | 0.153 | 0.249 | 99.8% |
| 30 | -0.033 | 0.027 | 0.160 | 0.239 | 99.4% | -0.033 | 0.027 | 0.161 | 0.253 | 99.7% |
| 35 | -0.030 | 0.027 | 0.161 | 0.240 | 99.7% | -0.029 | 0.027 | 0.162 | 0.255 | 99.9% |
| 50 | -0.038 | 0.034 | 0.180 | 0.240 | 98.9% | -0.036 | 0.034 | 0.181 | 0.267 | 99.5% |
| 75 | -0.054 | 0.062 | 0.244 | 0.240 | 94.3% | -0.042 | 0.063 | 0.248 | 0.322 | 98.6% |
| Abbreviation: CI, confidence interval; MCSD, Monte Carlo standard deviation; MSE, mean square error; SE, standard error  ^a^No missing data so neither approach was used | | | | | | | | | | |
